# pH-sensitivity of YFP provides an intracellular indicator of programmed cell death

**DOI:** 10.1186/1746-4811-6-27

**Published:** 2010-11-30

**Authors:** Bennett Young, Raymond Wightman, Robert Blanvillain, Sydney B Purcel, Patrick Gallois

**Affiliations:** 1 Faculty of Life Sciences, Michael Smith Building, University of Manchester, Oxford Road, Manchester M13 9PT, UK; 2 Biochimie et Physiologie Moléculaire des Plantes, CNRS UMR 5004, 2 place Viala, 34060 Montpellier Cedex 1, France

## Abstract

**Background:**

Programmed cell death (PCD) is an essential process for the life cycle of all multicellular organisms. In higher plants however, relatively little is known about the cascade of genes and signalling molecules responsible for the initiation and execution of PCD. To aid with the discovery and analysis of plant PCD regulators, we have designed a novel cell death assay based on low cytosolic pH as a marker of PCD.

**Results:**

The acidification that occurs in the cytosol during plant PCD was monitored by way of the extinction of YFP fluorescence at low pH. This fluorescence was recovered experimentally when bringing the intracellular pH back to 7, demonstrating that there was no protein degradation of YFP. Because it uses YFP, the assay is none-destructive, does not interfere with the PCD process and allows time-lapse studies to be carried out. In addition, changes of sub-cellular localisation can be visualised during PCD using the protein of interest fused to RFP. Coupled to a transient expression system, this pH-based assay can be used to functionally analyse genes involved in PCD, using point mutations or co-expressing PCD regulators. Transfecting *mBAX *and *AtBI-1*in onion epidermal cells showed that the pH shift is downstream of PCD suppression by *AtBI-1*. In addition, this method can be used to score PCD in tissues of stably transformed transgenic lines. As proof of principle, we show the example of YFP extinction during xylogenesis in *Arabidopsis*. This demonstrates that the assay is applicable to PCD studies in a variety of tissues.

**Conclusions:**

The observation that YFP fluorescence is lost during the plant PCD process provides a new tool to study the genetic regulation and cell biology of the process. In addition, plant cell biologists should make a note of this effect of PCD on YFP fluorescence to avoid misinterpretation of their data and to select a pH insensitive reporter if appropriate. This method represents an efficient and streamlined tool expected to bring insights on the process leading to the pH shift occurring during PCD.

## Background

PCD is a universal process across multicellular organisms that is highly regulated and tightly controlled by many genes. These genes are expected to act together to form organised cascades culminating in cell death. There are a few assays, which can be used to monitor PCD in plants in order to analyse the function and interaction of specific genes. These assays score PCD at various steps in the PCD process and each has its limitations. Some are destructive such as TUNEL assays for detecting the DNA fragmentation induced during PCD [1]. This assay involves fixing and permeabilising cells before labelling DNA fragment 3' ends using a terminal deoxynucleotidyl transferase and labelled dUTP (fluorescein, biotin, digoxigenin). Enzymatic assays for caspase-like proteases have become relatively prevalent in the plant literature as many synthetic caspase substrates are now commercially available [2]. Most chromogenic or fluorogenic substrates are based on a four amino acid peptide with has a higher affinity for a subset of animal caspases e.g. DEVD for caspase3 and 7. Typically plant protein extracts are buffered at pH 7 or 5.5 and incubated with one of the substrates at 50 to 100 μM. In addition, none destructive *in situ *caspase assay can be carried out using permeable caspase inhibitors coupled to the fluorescent molecule carboxyfluorescein [3]. The reagent easily permeates the cells and can irreversibly bind to caspase-like proteases inside the cell. Unbound inhibitor molecules are washed away to eliminate background. In addition, two types of cell permeable substrates have been used in pollen for *in vivo *studies of caspase activation [4]. Biotum^TD ^has developed a cell permeable substrate (Nucview 488) which when cleaved by caspase-like proteases releases a DNA dye which migrates to the cell nucleus and stains nuclear DNA [4]. The other substrate, CR(DEVD)_2_, is composed of two DEVD peptides coupled to the fluorophore cresyl violet (CR). Upon cleavage, the fluorescent CR marker is released as a red fluorescent product [4]. Recently, Zhang *et al*. 2009 [5] developed an *in vivo *PCD assay based on expressing a recombinant protein in cells that is a DEVD_FRET substrate cleaved by caspase3-like proteases. In this case, the fluorescence is lost when the substrate is cleaved. It requires costly confocal equipment. Finally, the dye mitotracker red can be used to detect a loss of mitochondrial membrane potential (ψmit) in cells undergoing PCD. A loss of ψmit has been reported e.g. in tobacco cells during heat-shock induced PCD [6] and during tracheary element formation [7]. This dye is a cationic lipophilic fluorochrome, which acts by accumulating in the negatively charged matrix of the mitochondria. The accumulation of this probe in the mitochondria is dependent upon the strength of the ψmit, the loss of which results in a proportional loss of mitotracker fluorescence [8].

Other generic live/dead assays used in PCD studies are non-destructive such as when using fluorescein diacetate (FDA) in an *in vivo *enzymatic assay. The assay relies on cellular esterase activity as a marker of live cells; the enzyme converts the non-fluorescent FDA to fluorescein [9]. Because FDA is used to score live cells, a time course of life to death cannot be generated in the same cell as FDA fluorescence persists in dead cells. Further assays are based on the loss of membrane permeability that occurs during PCD. The molecules Evans blue, sytox green and sytox orange are excluded from live cells and only diffuse into the cells when membrane permeability is compromised. Evans blue labels the whole cell blue while sytox green and sytox orange light up the nucleus after binding to DNA [10,11].

As plant vacuoles are acidic with a pH < 6 [12], it is logical to expect that vacuole rupture during PCD could result in cytosolic acidification. For example, authors of xylem differentiation studies had suggested that a cytosolic pH drop may occur when the vacuole ruptured during xylem PCD [13,14]. In addition, vacuole disruption was proposed as a defining characteristic for *bona fide *PCD as vacuole collapse was reported not to occur in necrotic cell death [15]. During PCD activated by self-incompatibility (SI) in the pollen tubes of *Papaver rhoeas, *Bosch *et al*. 2007 [4] were able to measure a dramatic drop of intracellular pH. Using a pH sensitive probe, they found that the intracellular pH of pollen tubes undergoing SI dropped from pH 7 to pH 5.5. A pH of 5.5 is remarkably close to the pH optima measured *in vitro *for most of the caspase-like activities detected during plant PCD [16], suggesting that lowering the pH may be part of the activation process for caspase-like activities in plants.

Evidence in the literature [4,14] and our own observation that YFP fluorescence was lost during the PCD process led us to consider that a large pH drop was a general feature of plant PCD and therefore a good candidate to develop a novel marker for PCD in plant cells.

## Results

### BAX induces PCD and intracellular acidification in onion cells

**Figure 1:**
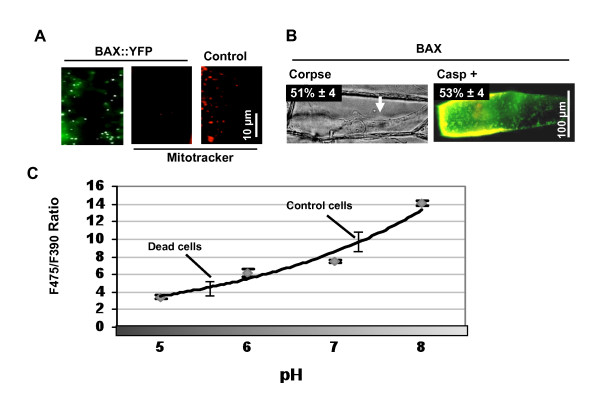
***BAX *expression induces PCD and intracellular acidification**. (A) Left panel; a close up of a *p35S:: BAX::YFP *expressing cell showing typical punctuated subcellular localisation. Centre panel; all BAX::YFP expressing cells were negative for mitotracker (n = 30). Right panel; untransfected cells were positive for mitotracker. Cells were observed 30 h post-bombardment. Scale bar 10 μm. (B) Corpse morphology (cellular collapse) and fluorescent VADase activity in cells expressing BAX 48 h post-bombardment. White arrow shows plasma membrane retraction. For VADase, unlabelled *p35S::BAX *was co-transfected with *p35S::RFP-td-tomato *in order to prevent overlap between the fluorescence of YFP and FAM-VAD-FMK. Inset panels show the percentage of BAX cells positive for these two PCD-hallmarks (values shown are minus the background obtained with an *RFP-td-tomato *control). Scale bar 100 μm. (C) Calibration curve for ptGFP readings *in vivo*. Cells were transfected with *p35S ptGFP *and incubated for 30 hours in the dark. Epidermal pieces were then incubated for 1 hour in 3 mL of buffer solution at various pHs with 0.003% Triton-20-X-100. F475/F390 ratiometric readings were then taken using 30 transfected cells for each pH value. Using the standard curve, the pH of control cells expressing ptGFP or of cells expressing BAX/ptGFP was interpolated using readings at 30 h. The average for all control cells or for all low pH BAX cells was plotted on the standard curve. Error bars are for 51 control cells and 15 low pH cells.

Transfection of onion epidermal cells using biolistics is a common technique used in many laboratories to examine the sub cellular localisation of proteins or to confirm protein-protein interactions using split YFP approaches [17-19]. Onion cells constitute an excellent experimental model, showing little background fluorescence compared to green tissues. To induce PCD and measure pH change in onion cells, we selected the mammalian pro-apoptotic gene *BAX*. There is no known homologue of *BAX *in plants but despite this, *BAX *induced hallmarks of PCD in tobacco plants [20] and in *Arabidopsis *[21,22]. *BAX::YFP *under the control of the 35S promoter was bombarded into onion cells. *BAX::YFP *has been shown to be targeted to mitochondria [22] and consistent with this, YFP fluorescence took a punctuate appearance in onion cells (Figure 1A). To ensure that BAX was capable of inducing *bona-fide *PCD in our system, induction of caspase-like protease activity, plasma membrane retraction and loss of mitochondrial membrane potential were investigated. At 30 hours, control cells treated with mitotracker showed a labelling distinctive of mitochondria while *BAX *expressing cells were all mitotracker negative (Figure 1A). Mitotracker is only labelling mitochondria that are functional and have a membrane potential (ψmit). A loss of ψmit is an early event in BAX and Nitric Oxide (NO) induced PCD in plants; it occurs as a result of the formation of the Permeability Transition Pore (PTP) [22,23]. In a separate experiment, *35S:: BAX *and *35S::td-tomato *were co::bombarded in onion cells. At 48 hours, caspase-like activity was detected *in situ *in 53% of transfected cells using a cell permeable, fluorescent pan-caspase inhibitor (FAM-VAD-FMK (Figure 1B). Induction of caspase-like activity is considered a strong hallmark for plant PCD [2]. In addition, 51% of transfected cell displayed a cell collapse with retraction of the plasma membrane from the cell wall and an increase in the prominence of the nucleus (Figure 1B). Such a cellular collapse, termed PCD corpse, has been shown to be specific to PCD as it is absent from plant cells that have died in an unregulated manner by necrosis [24]. The detection of these three PCD hallmarks confirmed that BAX induced PCD in onion cells.

To investigate the intracellular pH during PCD of onion cells, *Ptilosarcus GFP *(*PtGFP*) was chosen as a pH probe because of a broader pH-responsiveness and a greater acidic-stability than other pH probes such as pHluorins [25]. First, onion epidermal cells were bombarded with *ptGFP *under the 35S promoter, permeabilised and equilibrated using buffers at pH ranging from 5 to 8. Fluorescent ratiometric measurements (F475/F390) were taken. The calibration curve obtained (Figure 1C) fitted the first part of the sigmoidal curve described over a wider range of pH using recombinant *ptGFP *[25]. Next, 35S::*ptGFP *and 35S*::BAX *were co-bombarded to induce PCD and measure intracellular pH. After 30 hours of expression, all control cells expressing ptGFP only had an average intracellular pH of 7.3, a value typical for plant cell cytoplasm. By contrast at the same time point, cells co-expressing BAX and ptGFP had an acidic pH of 5.7 (Figure 1C). This pH value is consistent with the value of 5.5 reported by Bosch *et al*. 2007 [4] during PCD in pollen and is in support of using a buffer at pH 5.5 to develop a marker of the PCD process in plant cells.

### pH 5.5 attenuates the fluorescent signal of YFP *in vivo*

**Figure 2:**
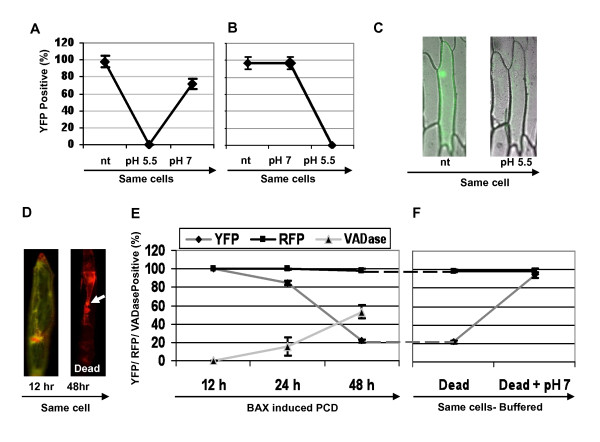
**The attenuation during PCD of YFP fluorescence is due to low intracellular pH**. (A, B) Epidermal cells were bombarded with *p35S::YFP *and incubated in the dark for 24 hours. YFP fluorescence was scored three times in the same onion epidermal cells, first untreated (nt) set as 100%, next buffered either at pH 5.5 or pH 7 and finally transferred to either pH 5.5 or pH 7. Modified intracellular pH was obtained by permeabilisation as in figure 1C. (C) Fluorescence images of one representative epidermal cell expressing *p35S::YFP *for 24 hours, first untreated (nt) and next buffered to pH 5.5; bright field and green channel have been merged. (D) Red and green merged images of the same cell co-expressing BAX::YFP and RFP-td-tomato. Before PCD (12 hr post-bombardment) both YFP and RFP fluorescence are detectable. After PCD (48 hr) only RFP fluorescence is detectable. White arrow shows plasma membrane retraction characteristic of PCD cell collapse. (E) BAX-induced PCD: the same cells co-expressing BAX::YFP and RFP-td-tomato were scored for YFP and RFP fluorescence at 12, 24 and 48 hours post-bombardment. Incubation with FAM-VAD-FMK allowed caspase-like protease activity to be detected in cells transfected with *p35S::BAX *(values shown are minus the background obtained with an *RFP-td-tomato *control). (F) At 48 hours, the same cells were buffered to pH 7 (as in A-B) and the YFP and RFP fluorescence was re-scored. Errors bars represent 2× SE of triplicates of 100 cells each.

It is known that YFP fluorescence can be reversibly attenuated at pH below 6, while RFP fluorescence is not affected [26-28]. To test if YFP could be used as an on/off pH indicator for scoring low pH during PCD *in vivo*, onion epidermal cells were transfected with a *35S::YFP *construct, permeabilised and submitted to various pH treatments (Figure 2A). The buffering treatment used is the same as the one used to establish the pH calibration curve in Figure 1. Buffers at pH 7 and pH 5.5 were chosen in order to mimic the pH drop measured in onion cells expressing BAX. The number of fluorescent cells in each sample was counted three consecutive times: 1) untreated, 2) after a first pH treatment and 3) after a second pH treatment (Figure 2A, B and 2C). A drop to pH 5.5 completely extinguished the fluorescence in expressing cells (Figure 2C) and when the same cells were subsequently buffered back to pH 7, a majority of cells recovered the YFP signal (Figure 2A). The pH 5.5 treatments therefore only attenuated the signal of YFP and did not permanently denature the protein. Since pH 7 as a first treatment had no effect on fluorescence (Figure 2B), it can be deduced that it is the pH and not the molarity of the buffer applied that had an effect on the fluorescence of YFP. By contrast and as expected, RFP was resistant to intracellular pH changes, as the pH 5.5 treatment had no effect on the number of RFP positive cells (data not shown). The above reconstruction experiments using buffers suggested that an intracellular pH drop from 7 to 5.5 in the cytosol should attenuate the fluorescence of YFP to the point that it became undetectable. This was not the case for the fluorescence of RFP, which could be used as a reference gene for transfection.

### PCD attenuates YFP but not RFP fluorescence *in vivo*

In order to test whether YFP fluorescence attenuation by low pH could be used to score PCD *in vivo*, we co-transfected cells with *RFP td-tomato *and *BAX *fused to *YFP *(*BAX::YFP*) (Figure 2D, E). The number of YFP and RFP expressing cells were counted at 12 h, 24 h and 48 h after bombardment (Figure 2E). At 12 h, every transfected cell was expressing both constructs and the number of BAX::YFP and RFP fluorescent cells was the same. At 24 h post-bombardment, the cells were re-counted and there was a small reduction (< 20%) in the number of cells fluorescent for both BAX::YFP and RFP. By the 48 h time point, there was an 80% reduction in the number of BAX::YFP fluorescent cells. For RFP, the number of fluorescent cells remained unchanged across all time points. To investigate the correlation between loss of YFP fluorescence and PCD, caspase-like protease activity was followed in cells expressing *BAX*. For this purpose, cells transfected with *35S::BAX *were incubated with FAM-VAD-FMK. The number of cells exhibiting caspase-like protease activity across the time points was found to correlate with the number of cells with an attenuated YFP signal (Figure 2E).

To confirm that the attenuation of YFP fluorescence observed in cells expressing BAX::YFP and RFP td-tomato (Figure 2D) was due to a pH drop and not to protein degradation, we buffered these cells to pH 7 for 1 hour. The lost BAX::YFP fluorescence was recovered in 95% of cells (Figure 2F). This confirmed that the pH drop measured during PCD is responsible for the loss of the YFP signal, while having no effect on RFP fluorescence.

### YFP can form the basis of a pH cell death assay to study the interaction of genes regulating PCD

**Figure 3:**
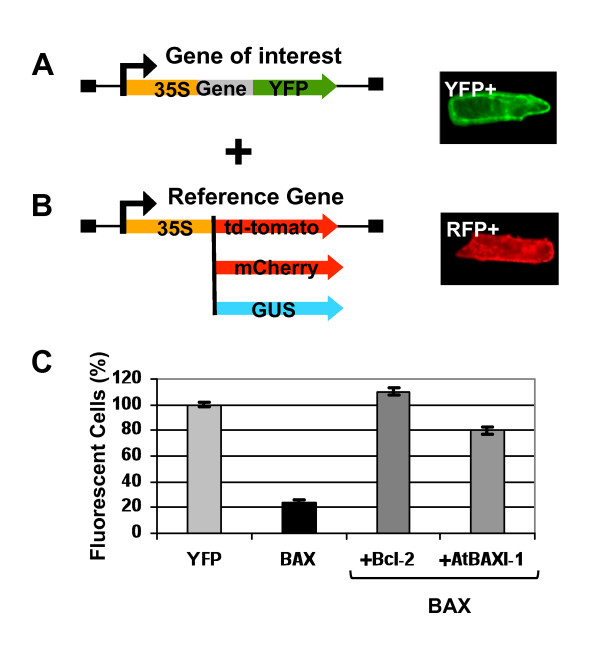
**Loss of YFP fluorescence induced by BAX can be prevented by AtBAXI-1 and Bcl-2**. (A) A typical construct for the gene of interest fused to YFP, the right panel shows an YFP expressing cell. (B) Various constructs for the reference gene. The right panel depicts a typical RFP td-tomato expressing cell. (C) Onion cells transfected using *p35S::YFP*, *p35S::BAX::YFP *(BAX), *p35S::BAX::YFP *(BAX) + *p35S::Bcl-2 *or *p35S::BAX::YFP *(BAX) + *p35S::AtBI-1 *(AtBAXI-1). *p35S::Bcl-2 *or *p35S::AtBI-1 *plasmids are co-transfected in a two-fold molar excess compared to *p35S::BAX::YFP. p35S::GUS *was co-transfected as the transfection reference. YFP positive cells are scored and expressed as a % of GUS expressing cells. Errors bars represent 2× SE of triplicates of 100 cells each.

Having shown that YFP can be used as a PCD marker, we set up a transient assay to study PCD genetic regulation. To illustrate the principle (Figure 3), a plasmid expressing a *BAX-YFP *fusion was bombarded in onion cells either on its own or co-bombarded with a two-molar excess of plasmid expressing a PCD suppressor. The suppressor genes used were previously shown to suppress PCD in plants: the human anti-apoptotic gene *Bcl-2 *[29] and the anti-PCD *Arabidopsis BAX-I *(*AtBI-1*) [21,22]. Finally, a *GUS *expressing plasmid was included in all bombardments to use GUS activity as a pH insensitive reference. Note that GUS histochemical assays are carried out using a buffered solution and therefore scoring GUS expression is not affected by the pH shift occurring during PCD. In this assay, every cell expresses both *YFP *and *GUS*. The number of YFP fluorescent cells is compared to the number of cells showing GUS enzymatic activity. 100% of GUS cells showing YFP fluorescence indicate no PCD. A percentage of YFP/GUS cells below 100% quantifies PCD in the population of transfected cells.

48 h after bombardment, cells with YFP fluorescence were counted. After scoring, the cells were incubated with X-GLUC to count cells with GUS enzymatic activity. The result is given in Figure 3C. Bombarding YFP as a negative control gave a 100% of YFP fluorescent cells. When expressing the fusion BAX::YFP, only 20% of GUS positive cells were YFP positive. This implied that 80% of transfected cells had lost YFP fluorescence 48 h after bombardment. This value obtained with *GUS *as a reference gene, is the same as the value obtained in previous experiments using the *RFP td-tomato *gene as a reference (see Figure 2E). *BAX::YFP *and *Bcl-2 *co-transfection gave 100% of YFP positive cells, whereas *BAX::YFP *and *AtBI-1 *co-transfection, gave 60% of YFP positive cells. The clear suppression effect of these two anti-PCD genes on the loss of YFP fluorescence in BAX::YFP expressing cells further demonstrated that the loss of YFP signal can be taken as a measure of PCD.

### Use of YFP to visualise PCD in whole root tissue

**Figure 4:**
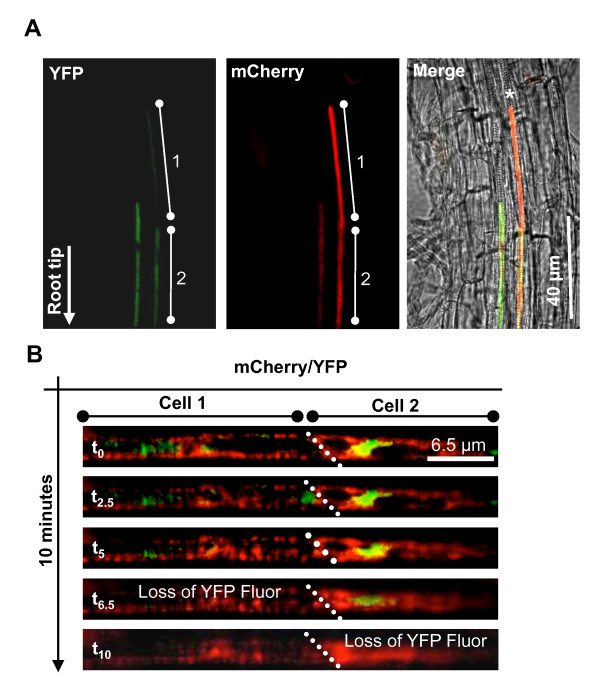
**Loss of YFP fluorescence during xylem development**. (A) Five day old *Arabidopsis *root expressing both YFP::CesA7 and mCherryER under the xylem specific promoter of the cellulose synthase subunit *IRX3*. White bars indicate two adjacent xylem cells (1 & 2). Left panel shows YFP::CesA7 fluorescence. Centre panel shows mCherryER fluorescence. Right panel shows both the YFP-CesA7 and mCherry ER fluorescence merged with the bright field image. * highlights the typical banding pattern characteristic of xylem. Images were captured using a YFP/mCherry dual (51019) filter. (B) Time-lapse photography of two adjacent xylem cells (1 & 2) in the same line as above. White dotted lines highlight the cell wall between cells 1 & 2. Green: YFP fluorescence; red: mCherry fluorescence.

As a proof of principle that our method allows the study of PCD in whole tissues, we show here YFP fluorescence attenuation during xylem differentiation in *Arabidopsis*. As explained in the introduction, the end point of xylem differentiation is PCD. Events occurring during the PCD of plant tracheary elements developing in zinnia cell culture can be examine using various microscopic and labelling techniques [13], including caspase substrates [30]. There is, however, no report of such observations with xylem cells embedded in the root tissue, as it has not been possible. To detect *in vivo *a possible pH drop in the xylem cell of intact roots of *Arabidopsis *seedlings, an *YFP::CesA7 *fusion and a *mCherryER *marker of the endoplasmic reticulum, both under the control of the cellulose synthase *IRX3 *promoter were introduced into *Arabidopsis *Ler. The *IRX3 *promoter is xylem specific and is expressed when secondary cell wall deposition starts [31]. Studies using zinnia culture have shown that PCD occurs after cell wall deposition [32]. Therefore, xylem specific expression of YFP at the time of cell wall deposition is expected to facilitate the observation of the onset of PCD during xylem development in intact roots. *mCherryER, *a pH insensitive RFP protein, has been introduced here to produce a stronger proof-of-principle experiment, but would not be required for routine scoring of cell death in tissues. Transgenic lines expressing both reporter genes were selected for a low/medium expression level with the fluorescent markers clearly observed deep within the root tissue. During the secondary-cell-wall deposition stage of xylem differentiation, both reporters were expressed and detected in the differentiating xylem only (Figure 4A). In Figure 4B, YFP and mCherryER are both seen expressed in two contiguous xylem cells. Previously, one of us (RW) has shown that the YFP::CesA7 protein fusion decorated transport vesicles in the cytoplasm, while mCherryER decorated the cortical ER [33]. mCherryER displayed a banding pattern typical of xylem cells during terminal differentiation (Figure 4A and 4B). As differentiation progressed away from the root tip, time-lapse photography of a file of two cells showed that in the cell distal to the root tip (Figure 4B, cell 1), YFP fluorescence was lost in the space of six minutes (Figure 4B and Additional File 1, Movie S1), while mCherry fluorescence remained unaffected and this for 40 minutes (data not shown). The cell proximal to the root tip (cell 2) started to lose YFP fluorescence five minutes after cell 1 (Figure 4B and Additional file 1, Movie S1). For a given cell, the whole sequence lasted *circa *five to six minutes. It should be noted that six minutes represent the time taken by a single cell to lose YFP fluorescence and not the time taken for the cell to reach that point after PCD initiation. By comparison, the PCD process including time for BAX gene expression can take up to 48 h in Figure 2.

## Discussion

We present here a method that uses YFP fluorescence as an indicator of the intracellular acidification that occurs during plant PCD. BAX expression in onion cells induced a loss of mitochondrial membrane potential, caspase-like activity and plasma membrane retraction, three hallmarks of plant PCD. We confirmed that the cytoplasmic pH drop from 7.3 to 5.5 described to occur during PCD in pollen tubes [4], occurred too in BAX-induced PCD in onion cells. We measured this pH change using the pH probe ptGFP, however this technique is too slow and labour intensive to score routinely PCD in plant cells. By contrast, we demonstrate here that the pH shift can be visualised much more simply as a loss of YFP fluorescence. The loss of YFP fluorescence correlated with induced caspase-like activity and could be inhibited by the PCD suppressors *AtBI-1*and *Bcl2*. PCD can therefore be scored as absence of YFP fluorescence in cell expressing YFP using a fluorescence microscope and appropriate detection settings.

YFP can be expressed in the cell of choice either on its own or as a fusion to a protein of interest to confirm expression, providing the fusion does not affect protein function. The ability to measure the consequence of gene expression in a none-destructive manner is an important tool to study gene function in any particular process. The use of YFP fluorescence as a PCD marker means that data can be captured in real time including information on sub-cellular localisation and kinetics. Conveniently, RFP fluorescence is not affected by changes in intracellular pH and remains detectable until the very last stages of PCD. This makes RFP proteins convenient references for transfection or gene expression. For example, sub-cellular localisation or expression can be monitored after the pH shift by fusing the gene of interest to the *RFP *sequence. The results section above shows examples using RFP td-tomato, RFP mCherry and GUS as transfection reference. Any reporter protein found to exhibit the required stability at low-pH would constitute a suitable alternative to these three proteins for use in our pH cell death assay.

As a demonstration of the usefulness of YFP as a PCD marker, we transiently expressed the mouse *BAX *gene to induce PCD in onion epidermal cells. Animal *BAX *has already been shown to induce PCD in plant cell [20]. Apoptosis induction in animal cells is linked with the localisation of BAX at the mitochondria, and the same BAX localisation is associated with PCD induction in plants [22]. *BAX-*induced PCD can be prevented by over expressing the plant PCD suppressor gene *AtBI-1 *[20,34]. We found *BAX *to behave in the onion assay exactly as predicted from the published studies above, reinforcing the proposition that data obtained using pH shift as a marker of PCD correlate with the results obtained with other PCD markers. In addition, we found the animal anti-apoptotic gene *Bcl2 *to block *BAX*-induced cell death in plants, possibly through direct physical interaction between the two proteins as described in animal cells [35,36]. Incidentally, these experiments showed that AtBI-1 and human Bcl-2 both suppressed PCD upstream of the pH shift, providing some insight in the cell death cascade in plants. Finally, we show here that onion cells are suitable for PCD studies. These cells come as a single layer of flat cells, facilitating microscopic observation. Onion epidermal cells are easy to handle and transfect using biolistics. We found that in addition to a pH shift, other PCD markers can be detected in onion cells such as caspase-like activities, loss of mitochondrial membrane potential and plasma membrane retraction.

In addition to experiments carried out using transient expression, we show the application of our method to the observation of differentiating xylem cells in *Arabidopsis *roots. PCD in differentiating xylem can be readily studied in *zinnia *cell culture [32] but not inside intact tissue such as root, as xylem cells are surrounded by several layers of cells. Our preliminary experiment suggests that YFP could be used to visualise PCD during the normal development of xylem. To our knowledge this is a first report using a PCD marker in live root and further work is required to characterised PCD in that experimental system. Nevertheless, our experiment demonstrates the power of cell-specific expression of YFP to visualise in real time and *in planta*, the pH shift marker of PCD. This approach could be extended to other developmental cell death systems.

## Conclusion

In conclusion, we show here that the fluorescence of cells expressing YFP is greatly reduced at a specific stage of PCD in plants. This observation is the basis of the PCD assay described here. Combined with gene co-expression systems, this assay provides a convenient tool to study both the genetic regulation and the cell biology of PCD. Additionally, because this loss of YFP fluorescence is a specific marker of pH during PCD, it can be used to bring insights in the pH shift process itself. Finally, cell biologists may be unaware of this effect of PCD upon YFP fluorescence, which could lead to misinterpretation of expression data. To avoid this, a pH insensitive reporter should be selected.

## Methods

### Plasmid DNA

pART7 with CaMV 35S and *Ptilosarcus *GFP (*PtGFP*) was bought from Nanolight, pinetop, USA. Mouse *BAX-alpha *fused to the a yellow fluorescent protein (*YFP) *[37] and *YFP *(pEYFP-N1 #6006-1 CLONTECH Laboratories, Inc.) clones were provided by A. Gilmore, Manchester UK, and cloned as Eco47-Xho1 fragments into pDH51 [38] cut with Sma1-Xho1. pDH51 provides a CaMV 35S promoter and terminator. A human *Bcl2 *cDNA clone was provided by T. Nishimoto, Kyushu Japan, and cloned as a BamH1-Sac1 fragment from pcDEB into pcGUS cut with BamH1-Sac1, providing a CaMV 35S promoter and NOS terminator. *AtBAX-Inhibitor1 *(At5g47120) was obtained from EST ATTS1836 via NASC, the European Arabidopsis Stock Centre, with the full coding sequence obtained by adding the sequence coding for MDAFSSF at the 5' end. The ORF was excised out of pBluescript SK+ into pDH51 using BamH1-Sal1. pRTL2-GUS expressing the beta-glucuronidase (GUS) gene is a gift from J. Carrington, College Station, USA. pAN57 containing *mannosidase-tdtomato *(RFP td-tomato) was obtained from Andreas Nebenführ [39]. All plasmid preps were performed using 'Nucleospin plasmid ' or 'Nucleobond Xtra midi plus' from Macherey-Nagel, Duren, BD.

### Bombardment Protocol

Each onion slice was transfected with a total of 10 μg of plasmid DNA. YFP and RFP plasmids were used in a 1:1 molar ratio. However, because GUS histochemical assays are more sensitive than YFP fluorescence detection, a dilution series of GUS plasmid concentration relative to YFP plasmid was carried out first to determine that a 1:1 ratio of cells expressing both GUS and YFP corresponded to a plasmid molar ratio of GUS 1:2.8 YFP. DNA was first precipitated onto 60 mg aliquots of 1.6 μm sterilised gold particles. Aliquots of gold particles were first vortexed for 30 seconds and then sonicated for 3 minutes. The DNA was then added in a volume under 80 μL, followed by 100 μL of 2.5 M CaCl_2 _and 20 μL of spermine 0.1 M with vortexing in-between each addition. The mix was further vortexed for 3 minutes and then left on ice for 15 minutes. The supernatant was removed and the particles were washed in 500 μL of ethanol. After 15 minutes on ice, the supernatant was removed and the particles were then washed again in ethanol this time using 200 μL. After one final 15-minute incubation on ice the supernatant was removed and the particles were re-suspended in 40 μL of ethanol. Bombardments were carried out on squares of onion fleshy scales of about 4 cm^2 ^and 3 mm thick, each onion square was mounted on 1% agar plates to hold it in position to be fired upon and to prevent drying. A 10 μL volume of particles was deposited onto the micro-projectile and left to dry before being used in the gun. Samples were fired using 1100 psi rupture discs in a PDS-1000/HE particle gun (BioRad) at a distance of 9 cm under a vacuum of -27 inches Hg (0.925 bar). Bombarded onion pieces were then kept epidermal side down on the agars plates, which were sealed with parafilm and kept at 23°C until use.

### YFP and RFP fluorescence

YFP positive cells and RFP positive cells were scored using a Leica DM5500 fitted with a Photometrics cascade II 512B EMCCD camera (Photometrics UK) and a dual filter YFP/dsRED (part 51019; Chroma Technology Corp.).

### Mitotracker staining for mitochondria depolarisation

Onion cells were incubated for five minutes in 100 nM mitotracker red CMX ROS (Invitrogen) and observed immediately using a fluorescence microscope and a dual filter YFP/dsRED (part 51019; Chroma Technology Corp.).

### *In situ *caspase activity

Activity was detected using the cell permeable and labelled Val-Ala-Asp peptide: FAM-VAD-FMK (APO LOGIX). Onion cells were incubated for 10 minutes in 4 μl of the 30× working dilution of the inhibitor diluted in 300 μl of water. Cells were washed twice in 5 ml of sterile distilled water before microscopic observation.

### Fluorescence ratio imaging of ptGFP

Fluorescence ratios were calculated in accordance to Schulte et al. (2006) [25]. Briefly, fluorescence images at excitation wavelengths of 475 nm and 390 nm were taken using a Nikon TE2000 PFS Widefield FRAP microscope fitted with a Cascade II EMCCD camera using a 10×/0.30 plan fluor objective. For this, cells expressing ptGFP were excited using a GFP filter (480/20) or a DAPI filter (402/15) with the emission wavelength centred on 540 nm. Images for each excitation were captured using Metamorph acquisition software from Molecular Devices. The ImageJ 1.42 free software was used to calculate a signal intensity value for every image. The ratio was subsequently calculated for the signal values obtained for an excitation at 475 nm and at 390 nm.

### Calibrating ptGFP signal

A calibration curve was created by incubating ptGFP-expressing cells in buffers at pH ranging from 5 to 8. For this, F475/F390 ratiometric readings were taken in epidermal cells expressing PtGFP and incubated in solutions buffered at a pH ranging from 5 to 8. For this, 30 hours after bombardment, onion epidermal cell peels transfected with *PtGFP *were incubated in 6 wells plates, each well containing 3 mL of pH-buffered solution and 0.003% Triton-X-100. Triton X-100 was added in order to permeabilise cell membranes and to facilitate equilibration with the extra cellular buffer. Buffers used were: sodium acetate 50 mM, pH 5, MES (2-(*N*-morpholino) ethanesulfonic acid) 50 mM, pH 6 and 7, and Tris-HCl (Tris (hydroxymethyl) aminomethane hydrochloride) 50 mM, pH 8. Onion epidermal peels were incubated in these solutions for 1 hour, with slow shaking before fluorescence analysis.

### GUS staining epidermal peels

Tissue samples were submerged in GUS staining solution (sodium phosphate buffer at pH 7, 10 mM EDTA, 0.1% Triton X-100, 2 mM potassium ferricyanide and 2 mM potassium ferrocyanide). For every one mL of this staining solution used, 1 μl of 5-Bromo-4-chloro-3-indolyl β-D-glucuronide (X-Gluc) (Melford, UK) was added from a stock at 50 mg/ml in dimethyl sulfoxide (DMSO). Samples submerged in the stain were vacuum infiltrated for 3 minutes, followed by 16 hours incubation at 37°C in the dark (plates were sealed with parafilm and then wrapped in tin foil to exclude light). The GUS stain solution was then removed and the tissue samples fixed in 70% ethanol.

### Fluorescent microscopy of roots

The constructs and the line expressing both the YFP-CesA7 fusion (Cellulose synthase subunit A7) and the mCherry endoreticulum (ER) reporter have been described in Wightman *et al*. 2008 [40]. Seedlings were grown in continuous light on vertical 0.5×MS salts (Duchefa), 1.5% agar plates. Root images were captured on a DMR microscope using an YFP/mCherry dual (51019) filter, a HCX PL APO CS ×63 water NA 1.2 objective and a SPOT Xplorer 4MP camera.

## Competing interests

The authors declare that they have no competing interests.

## Authors' contributions

BY participated to the conception the study, made constructs, carried out pH measurements, transient assays and drafted the manuscript. RW made constructs, transgenic lines and took xylem movies. RB conceived part of the study and carried out preliminary experiments, SP participated in pH measurements. PG conceived the study and participated in its design and coordination and drafted the manuscript. All authors read and approved the final manuscript.

## Supplementary Material

Additional file 1 **AVI Movie - PCD in xylem cells of *Arabidopsis *roots**. Three adjacent root cells expressing YFP::CesA7 and mCherryER under control of the xylem-specific promoter from the cellulose synthase subunit IRX3. *Arabidopsis *seedlings were grown for 5 days in continuous light on vertical 0.5 MS, 1.5% agar plates. Images were captured with a Leica DMR microscope using a YFP/mCherry dual (51019) filter, a HCX PL APO CS ×63 water NA 1.2 objective and a SPOT Xplorer 4MP camera. Click here for file
